# SHC1 Promotes Lung Cancer Metastasis by Interacting with EGFR

**DOI:** 10.1155/2022/3599832

**Published:** 2022-06-06

**Authors:** Pan Yang, Wei Li, Xiaoping Li

**Affiliations:** Department of Thoracic Surgery, Tianjin First Central Hospital, School of Medicine, Nankai University, Tianjin 300192, China

## Abstract

The study aims to explore the biological function of SHC1 in the development and progression of lung cancer. Meanwhile, the effect of SHC1 and EGFR on lung cancer was analyzed. The expression of SHC1 in lung cancer and adjacent tissues was analyzed by bioinformatics and immunohistochemistry. Meanwhile, the relationship between SHC1 expression and prognosis was analyzed. SHC1 overexpression and knockdown cell lines were constructed by overexpression plasmid and knockdown plasmid. Cell proliferation was detected by CCK-8. Cell invasion was detected by transwell. Apoptosis was detected by TUNEL. Interaction between SHC1 and EGFR was detected. The expression of SHC1 in lung adenocarcinoma tissues was significantly higher than that in paracancer tissues. Lung cancer patients with high SHC1 expression have a poor prognosis. The proliferation and invasion of SHC1 decreased with SHC1 knockout but increased after overexpression. EGFR may be a key interaction protein of SHC1. Overexpression of EGFR increases the oncogenic effect of SHC1. In conclusion, SHC1 plays a carcinogenic role in lung cancer. EGFR expression was significantly correlated with SHC1 and maybe a key interaction protein of SHC1. SHC1 interacts with EGFR to form a protein complex, which may be a new target for lung cancer metastasis.

## 1. Introduction 

Lung cancer is a serious threat to human life and health. Non-small cell lung cancer (NSCLC) accounts for 85% of all diagnosed lung cancers, and the overall 5-year survival rate of patients is less than 15% [1–3]. Metastasis and recurrence of tumor is one of the main causes of lung cancer treatment failure and death [4]. Therefore, finding new therapeutic targets is of great significance for the prevention and treatment of lung cancer [5, 6].

The proto-oncogene SHC family consists of SHCA, SHCB, SHCC, and SHCD4 gene members, which can encode 7 kinds of cohesive molecules [7, 8]. SHC family is widely expressed and functional. SHC can participate in the regulation of oxidative stress and various physiological functions through the phosphatidylinositol 3-kinase/threonine kinase signaling pathway and Ras-Raf-membrane receptor tyrosine protein kinase signaling pathway [8]. The SHC1 gene is located in zone 1, region 2 of human chromosome 1 [9]. SHC1 is believed to be involved in inhibiting neuronal apoptosis and regulating the receptor tyrosine kinase pathway [10]. With further study on SHC1, it has been found that the SHC1 gene plays an important role in the growth of breast cancer [11], gastrointestinal tumor cells [12, 13], and other tumor cells. However, the role of SHC1 in lung cancer needs further study.

Epidermal growth factor receptor (EGFR) is a receptor tyrosine kinase [14, 15]. After EGFR binding with ligand is activated, it can activate a variety of downstream signaling pathways, such as MAPK/ERK signaling pathway, PI3K/Akt signaling pathway, and STAT3 signaling pathway [16]. EGFR is closely associated with strong proliferation ability, poor differentiation ability, high lymph node metastasis rate, and poor prognosis of lung cancer [17–19]. A large number of studies have found that 40%–80% of NSCLC patients have abnormally high expression of EGFR or overactivation of the signal [20]. EGFR overexpression not only leads to resistance to EGFR-targeted drugs [21, 22] but also leads to tumor resistance to multiple chemotherapy drugs [23, 24]. Elucidating the EGFR regulation mechanism will help to search for new targeted molecules, formulate new therapeutic strategies, and provide an important theoretical basis for the development of new targeted drugs [25]. Through bioinformatics analysis, this study found that SHC1 may be associated with oncogenic protein EGFR. SHC1 may play a tumor-promoting role by regulating EGFR signaling pathway. To date, the relationship between SHC1 and EGFR has not been reported.

In this study, the mechanism of SHC1 in the development and progression of lung cancer was studied. To provide a theoretical basis for SHC1 as a therapeutic target of lung cancer, the purpose of this study was to determine the biological function and molecular mechanism of SHC1 in NSCLC. We first detected the expression of SHC1 in NSCLC tissues, and then studied the role and mechanism of SHC1 in cell proliferation, migration, invasion, cycle, and apoptosis. Through the expression changes of EMT pathway proteins, the functions of SHC1 and EGFR in lung development were explored, laying a foundation for clinical application.

## 2. Methods

### 2.1. Bioinformatics Analysis

Tumor expression profile data were downloaded from the TCGA database to analyze the expression of SHC1 in tumors. The expression levels of SHC1 in tumor tissues and adjacent normal tissues in the database were further compared. The immunohistochemical results were obtained from the Human Protein Atlas (http://www.proteinatlas.org/). Survival analysis of the SHC1 gene in lung cancer patients was performed using a GEPIA online tool (http://gepia.cancer-pku.cn/) to verify the relationship between SHC1 and prognosis.

### 2.2. Cell Culture

Lung cancer cells A549 and NCI–H446 were purchased from the cell bank of the Chinese Academy of Sciences, Shanghai. Medium 1640 and FBS were purchased from Gibco. Penicillin and streptomycin double antibody was purchased from Shanghai Biyuntian Company. Lung cancer cells were cultured in medium 1640 containing 10% of fetal bovine serum. The culture medium contained 1% of cyan-streptomycin double antibody. The cells were cultured in an incubator containing 5% of CO_2_ at 37°C. When the cells in the cell culture flask reached 80%, the cells were passed and the fluid was changed routinely.

### 2.3. Cell Transfection

Preexperiment was carried out according to the proportion of transfection reagent. Cells with good growth condition were selected, and 300,000 cells were added to each well of the six-well plate. The cell state was observed before transfection and transfected after the cell fusion degree reached 80%. The experimental group was A549 cells transfected with SHC1 plasmid, and the control group was A549 cells transfected with pcDNA3.1 plasmid. Plasmid was constructed from GenePharma (Guangzhou, China). Serum-free medium was used for transfection, and 1 × PEI was transfected with 2 *μ*g plasmid to 10 *μ*L. After 8 h, the medium was replaced with normal medium containing serum. After 24 h, the transfection efficiency was detected by qRT-PCR.

### 2.4. qRT-PCR

A549 experimental group cells transfected with SHC1 plasmid and A549 control group cells transfected with pcDNA3.1 plasmid were collected. Add 1 mL Trizol, shake, and mix well. The instructions of the cell RNA extraction kit to extract RNA were being followed. The cNDA synthesis kit operating instructions to reverse transcribe RNA to obtain cDNA were also being followed. The cDNA according to the relevant operating instructions of RT-PCR is then amplified. The instructions for use of the SYBR Premix EX Taq™ II real-time fluorescence quantitative PCR kit were referred. Real-time PCR reaction was performed using the ABI-PCR instrument, and each template was equipped with 3 replicate wells. The average CT value was taken and used 2^−ΔΔCT^ methods to express the relative expression of the SHC1 gene.

### 2.5. Transwell

A549 cells transfected with pcDNA3.1 plasmid and A549 cells transfected with SHC1 plasmid were suspended in serum-free medium in 24-well plates. Each of them is then added to a polycarbonate membrane in a transwell cell. The transwell membrane is coated with Matrigel. The membrane was covered with 500 *μ*L cell suspension. The submembrane medium was 750 *μ*L normal medium with serum. The cells were cultured in an incubator for 24 h, and then stained with 0.1% of crystal violet for 30 min. The crystal violet was cleaned with clean water and wiped the film clean with cotton swabs. Cell migration was observed and photographed under a fluorescence microscope.

### 2.6. TUNEL

The cell slides were immersed in 4% of paraformaldehyde PBS solution and fixed at room temperature for 20 min. It was rinsed with PBS solution, immersed in the sealing solution, and sealed at room temperature for 10 min. In the permeable solution (broken membrane solution), the ice infiltration was promoted for 2 min. 50 *μ*L TUNEL reaction mixture was added, covered with glass slide, and placed in a wet box. Reaction took place at 37°C for 60 min. DAPI staining was added for 30 min and rinsed with PBS. A pH value of 7.4 was dropped, 50% buffer glycerin, and the cover glass was covered. The fluorescence microscope was used to observe, and results were taken.

### 2.7. Cell Viability Was Measured by CCK-8

Logarithmic growing cells were collected. After counting, they were seeded into 96-well culture plates with 3 × 10^3^ cells/well. Incubate at 37°C and 5% CO_2_ for 72 h. Discard the original medium, and add 10 *μ*L CCK-8 and 90 *μ*L complete medium to each well. The cells were placed in an incubator and incubated for 1 h. The absorbance value at 450 nm wavelength (A) was detected by a microplate reader, and the cell viability was calculated.

### 2.8. Immunofluorescence

The cells were inoculated in a 12-well plate precovered with the cover glass. When the cell density was appropriate, the cells were cleaned with PBS solution. The cells were fixed with 4% of paraformaldehyde for 20 min and washed with PBS solution for 3 times. 0.5% Triton X-100 permeation for 20 min. It was cleaned with PBS solution for 3 times. 3% BSA was sealed at room temperature for 0.5 h. A primary antibody (1 : 100 dilution) was added and incubated overnight at 4°C. The cells were cleaned 3 times with PBS solution, 3 min each. The corresponding secondary antibody (diluted at 1 : 200) was added and incubated for 2 h at room temperature away from light. The cells were cleaned 3 times with PBS, 3 min each. The tablets were sealed with a tablet containing DAPI. The images were observed and photographed under a fluorescence microscope.

### 2.9. Subcutaneous Tumor Model of Nude Mice

All laboratory and animal care procedures are approved by the Animal Care and Use Committee of the Laboratory Animal Center. To construct an animal model of the xenograft tumor, transfected A549 cells growing to 70% were suspended in PBS, and each mouse was subcutaneously implanted with 1 × 10^6^ cells. The long and short diameters of the tumor were measured every two days after the cells were inoculated. The tumor size was measured with a vernier caliper. At the end of the 7th week, the nude mice were euthanized and subcutaneous tumor tissues were removed.

### 2.10. Immunohistochemical

The tissue specimens were fixed with 10% of neutral formaldehyde solution and treated conventionally. A continuous section of 4 *μ*m thick. Immunohistochemical staining was performed by the immunohistochemical SP method. The kit instructions were strictly followed. The primary antibody was mouse antihuman antibody (1 : 50, the R & D system). The second antibody was a biotin-labeled sheep antimouse antibody (1 : 1000, Beijing Zhongshan Jinqiao Biotechnology Co., LTD.). The known positive sections were used as positive control. PBS was used instead of primary antibody as negative control. It resulted in brownish-yellow granules in cytoplasm which were considered as positive protein. No yellow granules in cytoplasm is negative. Images were captured by an Olympus microphotography system.

### 2.11. Statistical Analysis

An IBM SPSS Statistics 26.0 software was used for data analysis. Statistical data were presented in the form of mean ± standard deviation. Two samples of measurement data were compared using an independent *T* test. Comparisons between multiple groups were analyzed using one-way ANOVA. Correlation analysis adopted two-variable correlation analysis. A univariate Cox regression model was used for survival analysis. *P* < 0.05 (bilateral) was statistically significant.

## 3. Results

### 3.1. High Expression of SHC1 in Lung Cancer Tissues Has a Poor Prognosis

**Figure 1:**
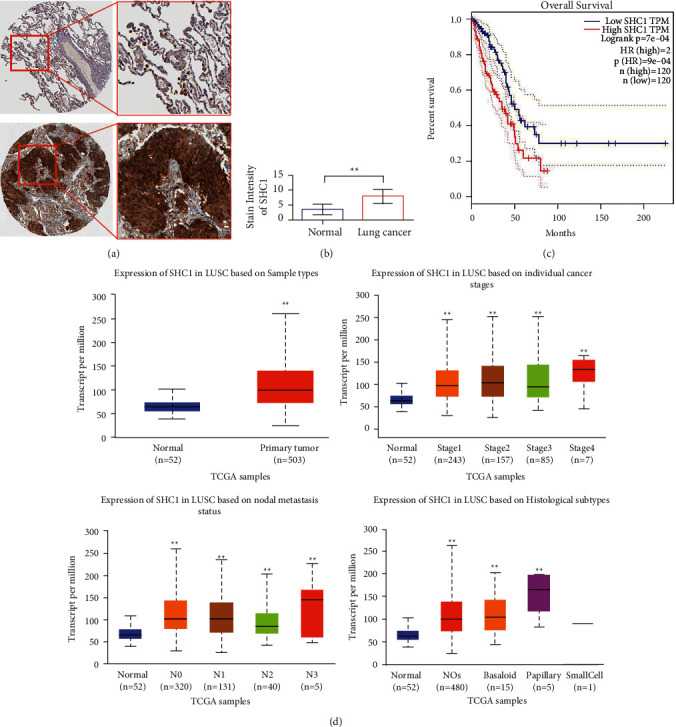
Identification and characterization of SHC1 in lung cancer. (a, b) Immunohistochemical detection of the SHC1 expression in adjacent tissues and lung cancer tissues. The immunohistochemical results were obtained from the Human Protein Atlas (http://www.proteinatlas.org/). (c) Survival analysis of SHC1 in lung cancer (high expression and poor prognosis). (d) UALCAN analysis. ^*∗*^*P* < 0.05,  ^∗∗^*P* < 0.01.

As shown in Figure 1(a), IHC results showed that SHC1 was significantly overexpressed in lung adenocarcinoma tissues compared with paracancer tissues. The results of statistical analysis showed that the high expression of SHC1 in lung cancer was statistically significant (Figure 1(b)). The GEPIA database was used to analyze SHC1 expression and overall patient survival in multiple tumors. As shown in Figure 1(c), SHC1 was highly expressed in lung cancer patients, and prognosis was poor (*P* < 0.01). Further, bioinformatics analysis showed that SHC1 was highly expressed in LUSC patients (*n* = 503) compared with normal tissue (*n* = 52) (*P* < 0.05). The higher the tumor stage and grade, the higher the expression of SHC1. In addition, SHC1 was highly expressed in different pathological types (Figure 1(d)).

### 3.2. Knockdown SHC1 Inhibits the Proliferation, Migration, and Invasion of Lung Cancer Cells and Induces Apoptosis

**Figure 2:**
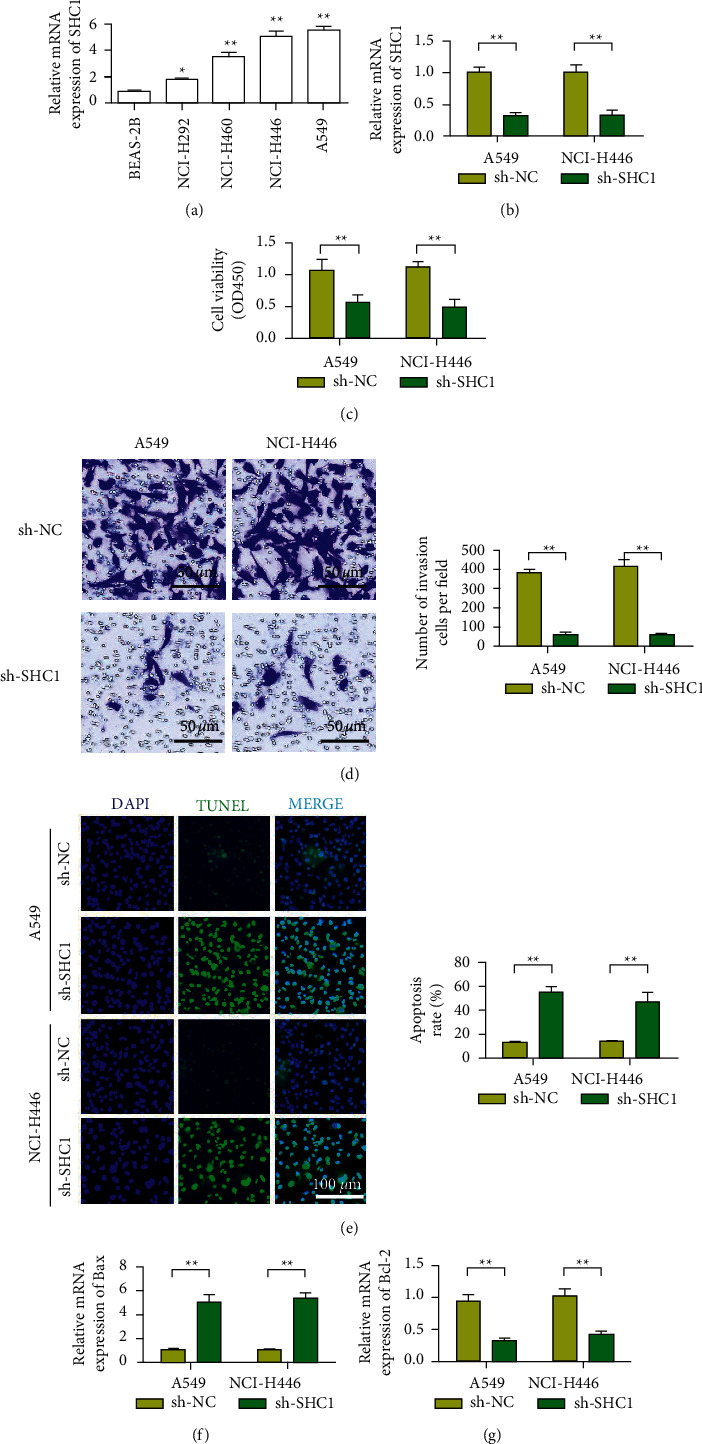
Knockdown of SHC1 inhibits the proliferation, migration, and invasion of lung cancer cells and induces apoptosis. (a) SHC1 expression is upregulated in lung cancer cell lines. (b) Using qRT-PCR to detect the expression changes of SHC1 in A549 and H446 transfected with shRNA and SHC1 cells. (c) CCK-8 analysis showed that downregulation of SHC1 inhibited DNA synthesis in A549 and H446 cells. (d) The transwell experiment, downregulation of SHC1 inhibited the invasion ability of A549 and H446 cells. (e) The TUNEL test results show that knocking down SHC1 promotes the apoptosis of A549 and H446 cells. (f) qRT-PCR detects the expression of Bax in A549 and H446 cells after knocking down SHC1. (g) qRT-PCR detects the expression of Bcl-2 in A549 and H446 cells after knocking down SHC1. Data are shown as the mean ± SEM; *n* = 3 independent experiments, ^*∗*^*P* < 0.05,  ^∗∗^*P* < 0.01.

Compared with human normal lung epithelial cells BEAS-2B, SHC1 expression was higher in lung cancer cell lines A549, NCI–H446, NCI–H460, and NCI–H292 (Figure 2(a)). In A549 and NCI–H446 cell lines, the expression of SHC1 in the sh-SHC1 group was significantly lower than that in the NC group (Figure 2(b), *P* < 0.01). As shown in Figure 2(c), in A549 and NCI–H446 cell lines, cell proliferation in the sh-SHC1 group was significantly lower than that in the NC group at 72 h (*P* < 0.01). Transwell results showed that the invasion ability of A549 and NCI–H446 cells transfected with sh-SHC1 plasmid was significantly weaker than that of sh-NC cells. The above results showed that SHC1 inhibited the invasion of LUNG cancer cells A549 (Figure 2(d)). To determine the effect of SHC1 on apoptosis, cell apoptosis was detected by TUNEL. It was found that the apoptosis rate of the transfected sh-SHC1 group was significantly higher than that of the transfected sh-NC group, and the difference between the two groups was significant (*P* < 0.01, Figure 2(e)). The results showed that silencing SHC1 could induce apoptosis. To investigate the mechanism of SHC1-induced apoptosis, we detected the expression of various apoptosis-related genes. The results showed that the expression of apoptosis-related gene Bax was upregulated after SHC1 silencing, while the apoptosis-inhibiting molecule Bcl-2 was downregulated (Figures 2(f) and 2(g)).

### 3.3. In A549 and NCI–H446 Cells, SHC1 Knockdown Inhibited EMT in Lung Cancer

**Figure 3:**
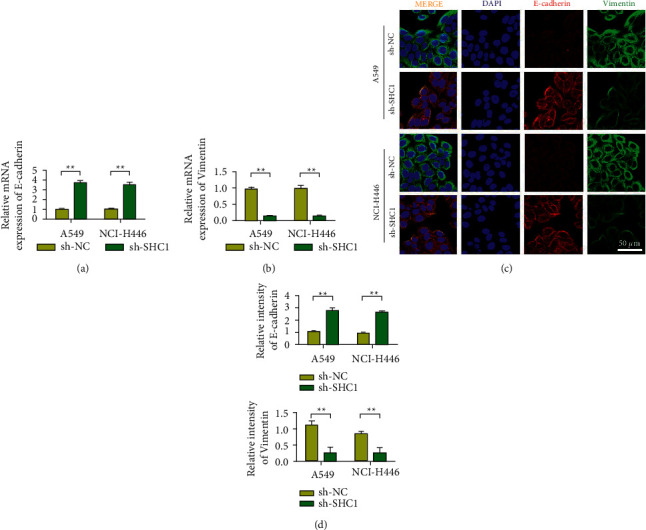
Knockdown of SHC1 inhibits lung cancer EMT in A549 and NCI–H446 cells. (a) In A549 and NCI–H446 cells, the expression of E-cadherin when SHC1 knockdown was detected by qRT-PCR. (b) In A549 and NCI–H446 cells, the expression of vimentin when SHC1 knockdown was detected by qRT-PCR. (c) In A549 and NCI–H446 cells, the expression of E-cadherin and vimentin during SHC1 knockdown was detected by immunofluorescence. (d) Statistical analysis results of the changes in the expression of E-cadherin and vimentin. Data are shown as the mean ± SEM; *n* = 3 independent experiments, ^∗∗^*P* < 0.01.

In A549 and NCI–H446 cells, the expression of E-cadherin during SHC1 knockdown was detected by QRT-PCR. The results showed that E-cadherin expression was upregulated after SHC1 silencing (Figure 3(a)). Detection results of vimentin expression showed that the expression of vimentin decreased after SHC1 silence (Figure 3(b)). In addition, the expression levels of E-cadherin and vimentin were detected by immunofluorescence. Immunofluorescence results were consistent with QRT-PCR, silencing SHC1 upregulated E-cadherin expression while inhibiting vimentin expression (Figures 3(c) and 3(d)).

### 3.4. Knockdown SHC1 Inhibits the Growth of Lung Cancer Tumors

**Figure 4:**
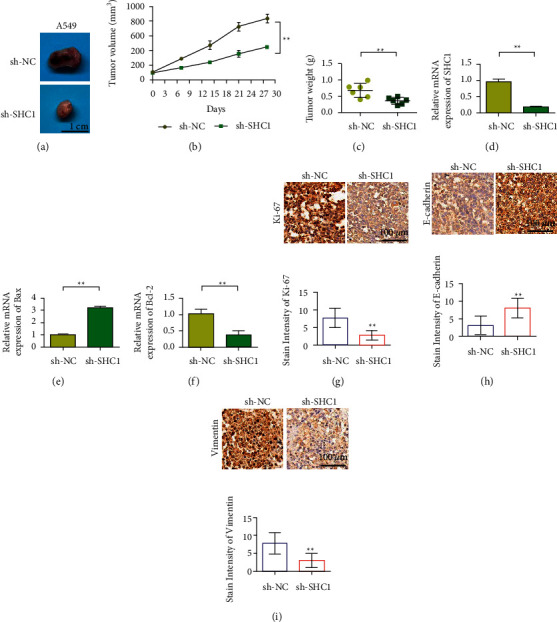
Knockdown of SHC1 inhibits the growth of lung cancer tumors. (a) Picture of subcutaneous transplantation tumor experiment. (b) Tumor volume curve graph. (c) Tumor weight detection. (d) qRT-PCR to detect the expression of SHC1 in tumor tissues. (e) qRT-PCR detects the expression of Bax in tumor tissues after knocking down SHC1. (f) qRT-PCR detects the expression of Bcl-2 in tumor tissues after knocking down SHC1. (g) The expression of Ki-67 was detected by immunohistochemistry. (h) Immunohistochemical detection of the E-cadherin expression. (i) Immunohistochemical detection of the vimentin expression. Data are shown as the mean ± SEM; *n* = 3 independent experiments, ^∗∗^*P* < 0.01.

**Figure 5:**
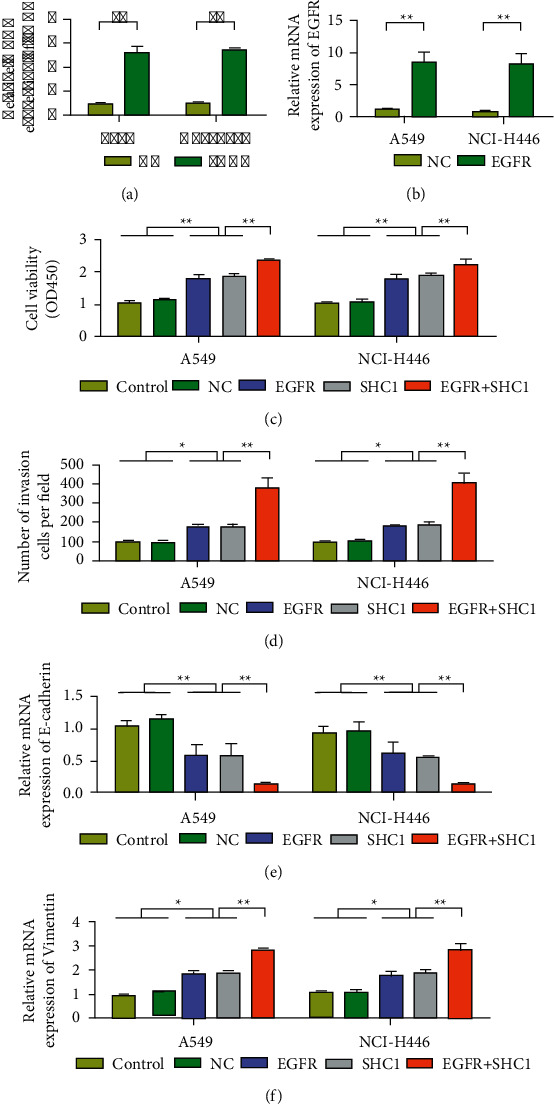
Overexpression of EGFR increases the cancer-promoting effect of SHC1. (a) Detection of SHC1 expression in NCI–H446 and A549 cells. (b) Detection of EGFR expression in NCI–H446 and A549 cells. (c) Detection of the cell proliferation rate of NCI–H446 and A549 cells. (d) Transwell detection of NCI–H446 and A549 cells. (e) Detection of the E-cadherin expression in NCI–H446 and A549 cells. (f) Detection of the vimentin expression in NCI–H446 and A549 cells. Data are shown as the mean ± SEM; *n* = 3 independent experiments, ^*∗*^*P* < 0.05,  ^∗∗^*P* < 0.01.

In order to further study the effect of SHC1 on lung cancer, we conducted animal experiments by subcutaneous tumor loading in nude mice. Animal experiment results showed that the proliferation rate of A549 cells transfected with sh-SHC1 plasmid was significantly slower than that of A549 cells transfected with sh-NC plasmid (Figures 4(a)-4(b)). Tumor results also showed that the tumor weight decreased after SHC1 silencing (Figure 4(c)). In tumor tissues, the expression level of SHC1 in the sh-SHC1 group was significantly lower than that in the NC group (Figure 4(d), *P* < 0.01). The apoptosis-related gene detection results showed that, after SHC1 silencing, the expression of Bax in tumor tissues increased, while the expression of Bcl-2 decreased (Figures 4(e)–4(f)). Subsequently, the expression of Ki-67 in tumor tissues was determined by immunohistochemistry. The results showed that Ki-67 expression decreased after SHC1 silencing (Figure 4(g)). Immunohistochemical results also showed that SHC1 silencing upregulated E-cadherin expression while inhibiting vimentin expression (Figures 5(h) and 5(i)). Inside tumor cells, protein complexes usually need to be formed to function. Protein interaction prediction results showed that EGFR might be the interacting protein of SHC1 (Table 1).

### 3.5. Overexpression of EGFR Increases the Oncogenic Effect of SHC1

**Figure 6:**
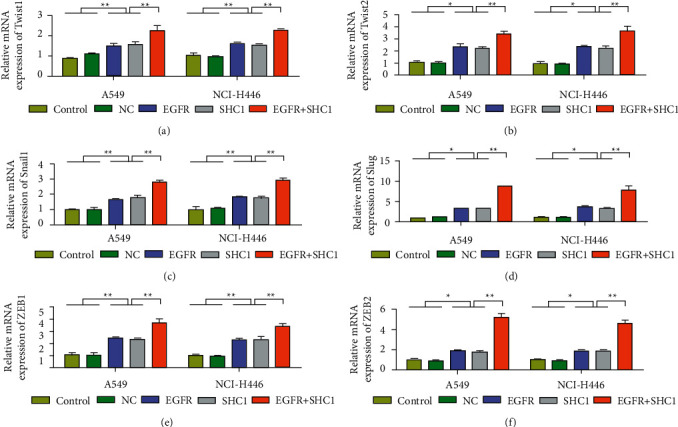
Overexpression of EGFR and SHC1 promotes the expression of tumor-related transcription factors. (a) In NCI–H446 and A549 cells, the expression of Twsit1 after overexpression of EGFR and SHC1 was detected by qRT-PCR. (b) In NCI–H446 and A549 cells, the expression of Twsit2 after overexpression of EGFR and SHC1 was detected by qRT-PCR. (c) In NCI–H446 and A549 cells, the expression of Snail1 after overexpression of EGFR and SHC1 was detected by qRT-PCR. (d) In NCI–H446 and A549 cells, the expression of Slug after overexpression of EGFR and SHC1 was detected by qRT-PCR. (e) In NCI–H446 and A549 cells, the expression of ZEB1 after overexpression of EGFR and SHC1 was detected by qRT-PCR. (f) In NCI–H446 and A549 cells, the expression of ZEB2 after overexpression of EGFR and SHC1 was detected by qRT-PCR. Data are shown as the mean ± SEM; *n* = 3 independent experiments, ^*∗*^*P* < 0.05,  ^∗∗^*P* < 0.01.

To further confirm that SHC1 promotes the proliferation and invasion of lung cancer cells through EGFR signaling, this study used SHC1 and EGFR expression plasmids to conduct experiments. Overexpression of pcDNA3.1-SHC1 cell line was successfully constructed, and the relative expression level of SHC1 in the OE group was significantly higher than that in the NC group (*P* < 0.01, Figure 5(a)). The relative expression level of EGFR was significantly higher in the transfected pcDNA3.1-EGFR group than in the NC group (*P* < 0.01, Figure 5(b)). The results showed that SHC1 or EGFR overexpressed in A549 and NCI–H446 lung cancer cells significantly increased proliferation and invasion capacity compared with the control group. On this basis, cells in the EGFR + SHC1 group had the highest proliferation and invasion ability (Figures 5(c)-5(d)). Meanwhile, after EGFR + SHC1 transfection, E-cadherin expression was decreased, while vimentin expression was increased (Figures 5(e)-5(f)). In addition, we also found that overexpression of EGFR and SHC1 promoted the expression of tumor-related transcription factors. QRT-PCR results showed that, after overexpression of EGFR and SHC1, the expression levels of Twsit1, Twist2, Snail, Slug, ZEB1, and ZEB2 in A549 and NCI–H446 cells were upregulated (Figure 6). These results suggest that SHC1 plays a tumor-promoting role through EGFR.

## 4. Discussion

The incidence and mortality of malignant tumors are increasing year by year [26]. Among them, lung cancer is in the first place in causing the death rate of malignant tumors [27]. Due to its poor prognosis and high mortality rate, it has brought great economic burden to the society [28].

SHC (Src homologue and collagen protein) gene family consists of SHC1 (SHCA), SHC2, and SHC3 (N-SHC) [29]. All SHC family proteins have unique domain pTB-CH1-SH2 structurally. PTB (phosphotyrosine binding) and SH2 (Srchomology2) are two phosphotyrosine binding regions [30]. Collagen homology 1 (CH1) contains tyrosine phosphorylation site, which is the signal output region of SHC protein [31]. As a typical junction protein, SHC1 plays an important role in transmitting receptor tyrosine protein kinase signals. SHC1 links to Ras pathways that activate receptor tyrosine kinases through GRB2/SOS. However, further research is needed to explore its mechanism in tumors. SHC1 is also involved in the regulation of oxidative stress, apoptosis, and senescence. Koch et al. [32] found that p66shcA can reduce the autologous antioxidant defense system of patients with alcoholic liver disease and promote the development of liver disease. SHC is highly expressed in early neural tissues [33] (mainly including neural stem cells and neural progenitor cells) and protects nerve cells by inhibiting apoptosis. Trinei et al. [34] pointed out that p66SHCA was proved to be a downstream molecule of p53 in the process of cell apoptosis induced by external oxygen stress and played an important role in its mediated apoptosis process. SHC1 was selectively upregulated in the liver and brain of aged rats. These results suggest that SHC promotes normal cell senescence [35]. In recent years, the relationship between overexpression of SHC and carcinogenesis has gradually attracted people's attention. SHC1 (SHCA) is the most prominent one. Some researchers have found high levels of SHC in thyroid cancer cells [36]. Veeramani et al. [37] found that SHC1 seems to have a role in promoting proliferation in tumor cells. Currently, there are few studies on the expression and biological effects of SHC1 in lung cancer.

This study comprehensively summarized the expression of SHC1 in tumors. High expression of SHC1 was found in lung cancer. The effect of SHC1 on overall survival was also analyzed. The results showed that differential expression of SHC1 influenced the overall survival of lung cancer patients. Therefore, the SHC1 gene in lung cancer has been studied in depth. First, SHC1 knockout lung cancer cell lines were constructed. Subsequently, experiments related to the cell proliferation, migration, and invasion were carried out. The results showed that silencing SHC1 inhibited the proliferation and invasion of lung cancer cells. The trend of EMT-related markers showed that SHC1 silencing could inhibit EMT in lung cancer cells. In order to further study the mechanism of SHC1, protein-protein interaction prediction was performed by FpClass. Finally, by predicting the protein interaction network involved in SHC1 in the database, we found a clear interaction between SHC1 and EGFR.

A large number of studies have found that high expression or abnormal activation of EGFR is closely related to the occurrence, development, recurrence, metastasis, and drug resistance of lung cancer [38]. EGFR tyrosine kinase inhibitors (EGFR-TKIs) have been widely used as first-line drugs in patients with non-small cell lung cancer with EGFR mutation [39, 40]. But the long-term effects are less than satisfactory. The main reason is that some tumor patients are not sensitive to EGFR-targeted therapy [41]. It was found that, in addition to acquiring drug-resistant mutations, the continued activation and transmission of EGFR signals provided survival advantages for cancer cells. It also increases drug resistance of tumor cells [42]. Based on this, it is necessary to find new strategies to target EGFR for the lung cancer treatment. This study found that SHC1 and EGFR may be interacted. Knocking SHC1 down attenuates its interaction with EGFR. Therefore, targeted interference with SHC1/EGFR interactions may be a new strategy for the lung cancer treatment.

In this study, SHC1 was significantly associated with EGFR at both clinical and cellular levels. EGFR showed a corresponding trend at the cellular level with the change of SHC1. EGFR may be a downstream regulatory protein of SHC1 interaction. However, the specific mechanism by which SHC1 interacts with EGFR remains unclear. Next, we will study the relationship between SHC1 and EGFR by the Duolink PLA experiment. In addition, how EGFR affects SHC1 function needs further study. In conclusion, SHC1 and EGFR are expected to become new therapeutic targets for lung cancer.

## Figures and Tables

**Table 1:** Protein-protein interaction between SHC1 and EGFR (SHC1 interaction protein prediction).

Query ID	Predicted partner symbol	Predicted partner UniProt	Total score
SHC1	EGFR	P00533	0.9429
SHC1	AXL	P30530	0.8826
SHC1	IL2RG	P31785	0.8826
SHC1	FCGR2B	P31994	0.8826
SHC1	CSF2RB	P32927	0.8826
SHC1	HSPA4	P34932	0.8826
SHC1	IRS1	P35568	0.8826
SHC1	FLT4	P35916	0.8826
SHC1	KDR	P35968	0.8826
SHC1	STAT3	P40763	0.8826

## Data Availability

The data used to support the findings of this study are available from the corresponding author upon request.
